# Role of SimMan in teaching clinical skills to preclinical medical students

**DOI:** 10.1186/1472-6920-13-20

**Published:** 2013-02-10

**Authors:** Meenakshi Swamy, Thomas C Bloomfield, Robert H Thomas, Harnaik Singh, Roger F Searle

**Affiliations:** 1 School of Medicine and Health, The Holliday Building, Durham University Queen's Campus, University Boulevard, Stockton on Tees, TS17 6BH, UK; 2 Royal Infirmary of Edinburgh, Old Dalkeith Road, Edinburgh, EH16 4SA, UK; 3 University Hospital North Tees, Hardwick Road, Stockton-on-Tees, TS19 8PE, UK; 4 Freeman Hospital, Freeman road High Heaton, Newcastle upon Tyne, NE7 7DN, UK; 5 Anatomy and Clinical Skills, School of Medical Sciences Education Development, The Medical School, Newcastle University, Newcastle upon Tyne, NE2 4HH, UK

## Abstract

**Background:**

Simulation training has potential in developing clinical skills in pre-clinical medical students, but there is little evidence on its effectiveness.

**Methods:**

Twenty four first year graduate entry preclinical medical students participated in this crossover study. They were divided into two groups, one performed chest examination on each other and the other used SimMan. The groups then crossed over. A pretest, midtest and post-test was conducted in which the students answered the same questionnaire with ten questions on knowledge, and confidence levels rated using a 5 point Likert scale. They were assessed formatively using the OSCE marking scheme. At the end of the session, 23 students completed a feedback questionnaire. Data was analyzed using one-way ANOVA and independent *t*-test.

**Results:**

When the two groups were compared, there was no significant difference in the pretest and the post-test scores on knowledge questions whereas the midtest scores increased significantly (P< 0.001) with the group using SimMan initially scoring higher. A significant increase in the test scores was seen between the pre-test and the mid-test for this group (P=0.009). There was a similar albeit non significant trend between the midtest and the post-test for the group using peer examination initially.

Mean confidence ratings increased from the pretest to midtest and then further in the post-test for both groups. Their confidence ratings increased significantly in differentiating between normal and abnormal signs [Group starting with SimMan, between pretest and midtest (P= 0.01) and group starting with peer examination, between midtest and post-test (P=0.02)]. When the students’ ability to perform examination on each other for both groups was compared, there was a significant increase in the scores of the group starting with SimMan (P=0.007).

**Conclusions:**

This pilot study demonstrated a significant improvement in the students’ knowledge and competence to perform chest examination after simulation with an increase in the student’s perceived levels of confidence. Feedback from the students was extremely positive. SimMan acts as a useful adjunct to teach clinical skills to preclinical medical students by providing a simulated safe environment and thus aids in bridging the gap between the preclinical and clinical years in medical undergraduate education.

## Background

The General Medical Council’s document, Tomorrow’s Doctors [1], called for streamlining of the curriculum, with a need for enhanced clinical skills training in the undergraduate medical education in order to prepare effective doctors. In response to these recommendations, clinical skills training has gained greater importance over the years [2,3] and runs alongside theoretical learning to seek synergy between components of learning in UK medical schools. This has led to a drive towards the development of clinical skills learning facilities where protected time can be devoted for practicing a wide range of skills in a safe environment. The focus has been on smoothing their transition from being a student to becoming a doctor.

At Newcastle University, clinical skills training starts at the outset of year 1 for the undergraduate medical students, comprising of clinical examination, procedural skills and communication skills. They are introduced to generic clinical skills in such a way that they are able to recall, apply and integrate the relevant theoretical knowledge. The students practice clinical skills under direct supervision, receive immediate feedback on their performance and thus acquire clinical skills required of them in order to subsequently be able to perform them on patients.

However, due to limited clinical exposure to patients, it is not only challenging for medical students in Phase 1 Medicine (pre-clinical years) to systematically learn and develop clinical skills appropriate to working in a clinical environment, but also to be able to apply these skills when they move onto their clinical rotations in Phase 2 Medicine (clinical years). The transition from preclinical to clinical training is huge for the students and several studies have documented that the transition is quite stressful [4].

Medical simulation is considered to bridge the gap between the classroom and clinical environment. In the last two decades, medical education seems to rely more on simulation technology to help young doctors acquire clinical skills effectively [5]. Simulation enables learners, from novice to expert to practice and develop clinical skills without any fear of harm to patients. Other benefits include increase in retention and accuracy; allows repetition and can be tailored to individuals [6]. Although it does not duplicate educating students with real patients in a genuine clinical setting, evidence suggests that it complements learning and thus can be used to prepare students for real patient contact [5].

Medical simulators range from simple replications of body parts for task based learning of some examination skills, to more sophisticated high fidelity patient simulators driven by complex pathophysiological computer models which are developed to replicate clinical environments. They are being used to teach cognitive strategies, crisis resource management, professionalism and teamwork for undergraduates and high student satisfaction has been reported by its users [7]. Its effectiveness as a teaching tool is well established by various studies [8]. Laerdal SimMan, a moderate fidelity patient simulator is now widely used in medical education [6].

Preclinical medical students at Newcastle University practice clinical skills on each other and become familiar with what is normal in a safe and supportive environment, the clinical skills laboratory, which is a simulated ward. We have used Laerdal SimMan 3G, in the clinical skills laboratory to reproduce clinical settings as close as possible and to provide students with an opportunity to recall, select and sequence the acquired information and experience differentiating between normal and abnormal signs on SimMan.

The aims of the study were to ascertain if SimMan could be used as an adjunct to facilitate student’s ability to acquire clinical skills by determining the effect of SimMan on students’ knowledge and confidence levels, formatively assessing their performance and evaluating the use of SimMan during clinical skills teaching in Phase 1 medical undergraduate programme.

## Methods

### Participants

Twenty four first year graduate entry preclinical medical students from the Accelerated MBBS programme at Newcastle University, UK were included in this study.

### Simulator and setting

We used Laerdal SimMan 3G, a wireless life size patient manikin that can talk with pre-recorded sounds and speech, breathe with normal and abnormal breath sounds and produce heart sounds, palpable pulses and unilateral/bilateral chest movements. It is connected to a monitor which displays parameters such as oxygen saturation, ECG trace, pulse rate and blood pressure. These parameters were controlled by the computer. As SimMan can be programmed with a range of clinical examination findings, in our study it was programmed to display up to three abnormal signs in each of the four selected respiratory and cardiovascular clinical conditions based on students’ case driven blended problem based learning (PBL) curriculum (Table 1).

**Table 1:** Clinical conditions with corresponding signs used in the study

**Clinical conditions**	**Signs**
Acute asthma	Increased respiratory rate and wheeze
Acute COPD	Crepitations, wheeze, and cyanosis
Aortic stenosis	Ejection systolic murmur and basal crepitations
Infective endocarditis	Murmur and splinter haemorrhages

Two SimMan 3G were set up on a standard hospital bed equipped with monitors, simulated oxygen supply and the usual supplies found on the ward, in the clinical skills lab which has a structured layout similar to the old Nightingale wards with curtained cubicles.

All the students had been previously taught the clinical skill of respiratory and cardiovascular system examination on each other. The learning outcomes were stated for the students and the tutors.

### Study design

The students were divided into two groups of twelve (A and B). They were then further subdivided into subgroups of 3 students who were facilitated by a tutor (medical doctor) for one hour with thirty minutes spent on examining each other and the other thirty minutes on SimMan (Additional file 1: Appendix1). During this session, every student performed respiratory or cardiovascular system examination for five- seven minutes on each other or on SimMan and received feedback after their performance from the tutor for 3–5 minutes. In majority of the cases, complete time allocated was utilized. A pre-test was conducted at the beginning of the session. The students of both groups completed a questionnaire which consisted of ten questions based on knowledge and confidence levels rated using a 5-point Likert scale (1-very low to 5-very high). Then students performed respiratory and cardiovascular examination on each other (Group A) or on SimMan (Group B). A mid-test (with the same questionnaire) was conducted. The groups then crossed over with the students in the group A performing respiratory and cardiovascular examination on SimMan while students in group B performed respiratory and cardiovascular examination on each other. A post test (with the same questionnaire) was carried out. The cross over study was undertaken to provide equivalent learning opportunities for all the students. Hence every student performed either cardiovascular or respiratory system examination on each other as well as on SimMan.

The students were observed and scored by the tutor individually in the subgroup while performing examination on SimMan or on each other using a scoring scheme which is used to assess them during OSCE. Two extra points in addition to the twenty marks on the scoring scheme were allocated for identifying abnormal signs on SimMan (1 mark if the student identified that there were abnormalities but was unsure of what they were and 2 marks if the student correctly identified the abnormalities).

At the end of the session, 23 students (96%) completed a feedback questionnaire about the entire session.

Ethical approval was obtained from Faculty of Medical Sciences Ethics Committee, Newcastle University (Reference number - 00552/2012).

### Data analysis

Anonymized data was analyzed using SPSS version 19 (SPSS Inc., Chicago, IL). The specific statistical analysis is described within the results for each stage of the study. A value of P < 0.05 was considered statistically significant for all the tests.

## Results

Twenty four students participated in the study.

### Crossover study

The knowledge test scores (Table 2) did not differ significantly between group A and group B for the pretest (independent *t*-test, t = 0.618, P = 0.543). This shows that that the baseline level of knowledge of both groups was equal. The knowledge test scores increased significantly between the two groups for the midtest (independent *t*-test, t = 4.284, P = < 0.001), group B after performing the examination on SimMan scored higher when compared to group A. There was no significant difference in the post-test knowledge scores between the two groups (independent *t*-test, t = 0.965, P = 0.347) which demonstrates that after both groups had performed examination on SimMan, their knowledge had improved equally.

**Table 2:** Knowledge test scores of the students in the groups A and B

**Tests**	**Groups**	**Mean**	**Standard deviation**
**Pretest**	**A**	**5.66**	**1.07**
	**B**	**5.91**	**0.90**
**Midtest**	**A**	**5.66**	**0.70**
	**B**	**6.75**	**0.45**
**Post- test**	**A**	**6.11**	**0.60**
	**B**	**6.41**	**0.79**

Statistical analysis (one-way ANOVA with bonferroni correction) demonstrated that there was a significant difference between the pretest and midtest scores of group B (F= 3.853, P= 0.031). Further analysis (independent *t*-test) demonstrated that there was a significant increase in the midtest scores (after performing examination on SimMan) when compared with the pretest scores in the group B (independent *t*-test, t = 2.865, P = 0.009). There was a similar non significant trend in group A with an increase in the post- test scores (after performing examination on SimMan) when compared with midtest scores (independent *t*-test, t = 1.437, P = 0.170). Thus students scored higher in the knowledge test after examining SimMan when compared to students who performed the examination on each other.

Although the mean confidence in performing respiratory/cardiovascular examination increased from the pretest to midtest and then further in the post-test for both the groups, there was no statistical difference between the two groups, A and B. After performing examination on SimMan, both groups felt that they were more confident in differentiating between normal and abnormal signs [group B, pretest(2.25± 0.75) vs midtest (3± 0.60), independent *t*-test, t = 2.458, P = 0.01) and group A, midtest (2.75± 1.05) vs post test (3.58 ± 0.51), independent *t*-test, t = 2.691, P = 0.02)].

### Formative assessment

Although there was an increase in performance scores in both groups when they performed for the second time (examining either each other or on SimMan) this was not statistically significant (Table 3). The students in group B following their SimMan experience scored significantly higher when compared to those in group A for performing respiratory/cardiovascular examination on each other [group B, 15.33(± 2.05) vs group A, 12.25(± 2.89), independent *t*-test, t = 3.006, P = 0.007].

**Table 3:** Formative exam scores

	**Group A (Mean)**	**Group B (Mean)**	
**Peer examination**	**12.25**	**15.33**	**P= 0.007**
**SimMan**	**14.16**	**15.16**	**P=0.47**

### Evaluation

Responses were received from 23(96%) students. All students strongly agreed that the session was useful. Some of the free text comments included “Was useful to identify areas of weakness in my technique”, “Feel more confident in future about learning abnormal signs and to face exams”, “Good practice before OSCE on SimMan as it helps to remember the sequence”, “SimMan is scary and challenging but makes you think. It has improved my confidence”, “Helps to reaffirm my knowledge”, “Strongly reinforced examination procedure and gave us the opportunity to see abnormal signs”, “Useful to practice but also good to have a focus on next year. It is easy to be overly focussed on exams and this counts on that”, “Really good to find out where I am at”, “Good revision, learnt new things about pathology”, “Supervised revision in small groups and specific feedback was useful. We were able to ask all questions on our minds”.

For 18 (78%) of 23 students, the session improved their appreciation of the importance of acquiring clinical skills and 3 (13%) were unsure. They all enjoyed the session and found the use of SimMan and the feedback given was useful. 22 (96%) students felt that it made them more prepared to examine real patients with one of them being unsure.

## Discussion

In the preclinical phase of undergraduate medical training at Newcastle University, medical students are taught to perform cardiovascular and respiratory examination on each other (peer examination) to help them appreciate normal findings. The advantage is that the students are practicing on a live person but the disadvantage is that they are aware that they are examining a normal healthy person. In order to bring in the variability and help students to actually look for normal and abnormal signs rather than just comment that they were either present or absent, students’ were asked to examine SimMan.

In this pilot study, we compared two clinical skills teaching modalities, peer examination and examination using SimMan. It was found that students’ knowledge required to perform cardiovascular and respiratory system examination did not improve by repetition of peer examination but their knowledge improved considerably after they performed examination on SimMan when compared to peer examination. This could be because SimMan provided the students with an opportunity of learning to identify abnormal findings and thus reiterating the knowledge required to identify normal findings. SimMan was as effective as peer examination in increasing the students’ confidence. Our findings are similar to those of Halm et al^11^, who found that SimMan increased students’ toxicology knowledge and self confidence in an emergency department setting prior to beginning clerkship experiences.

It is essential at this stage of their medical school training, that students perform clinical examinations on each other to become familiar with normal findings so that they recognise any deviation from the normal in patients in their clinical years. SimMan when used as an adjunct provides students with an opportunity to differentiate normal from abnormal findings in a simulated safe environment repeatedly without any harm to the patients and thus can aid in reinforcing normal findings. We found that students’ confidence increased significantly for differentiating between normal and abnormal clinical signs after simulation as they have had an opportunity to appreciate both normal findings and deviation from normal which is what they are likely to encounter in clinical practice. In our study, twenty one students recognised the abnormal signs and among them, nine students identified what they were.

When student’s ability to perform cardiovascular/respiratory examination on each other was compared before and after simulation, it was found that the students scored significantly higher after simulation. The finding that Group B scored better on peer examination could be explained by the fact the they had more training (SimMan and Peer examination) than Group A which had been exposed only to peer examination at the time of the formative exam. However, when the results are analysed in further detail, the scores of the students in Group B who performed examination on SimMan first were higher when compared to Group A who performed peer examination initially. After midtest, there was no statistically significant difference between the two groups suggesting the scores of Group A improved vastly to match Group B at the end of the session when both had completed cardiopulmonary examination on SimMan. Thus SimMan can help students to improve their skills during cardiovascular and respiratory examination. Smaller group size of about three with involvement of all the students also proved to be beneficial.

Other studies have shown positive evaluations for the use of Laerdal SimMan to teach undergraduate medical students in the context of management of medical emergencies [8-10]. This was confirmed by our study suggesting that SimMan can be a valuable tool for teaching clinical skills such as cardiovascular and respiratory examination for preclinical medical students.

SimMan not only provides students with an opportunity to apply their integrated knowledge and perform clinical skills on a simulated patient but also helps them to appreciate the significance of acquiring basic science knowledge required to perform these skills. Godden and Baddeley [11] tested recall in two contexts, on land and underwater; and suggested that learning and recall is context dependant. Thus learning actively in a clinically realistic environment may promote retrieval of the information when required in future clinical practice. The clinical relevance is obvious to the students and hence helps them to focus on their future clinical practice.

There are other options like Harvey’s simulator, ventriloscope and standardised patients available for the students to appreciate and practice examination of various systems. Each of them have their own pros and cons. SimMan while being expensive, has the advantage of providing the students with a more realistic and holistic experience of examining and treating a patient in a safe simulated environment with repetition at their own convenience. It has the benefits of wireless connectivity, ability to replicate chest movements/cyanosis, monitors showing parameters like oxygen saturation/respiratory rate which respond to the interventions, etc. Standardised patients probably provide the best replication of a real case scenario but have the problems of availability, accessibility, feasibility for a large group of students to practice and expenses in the long run. Use of patient simulators is becoming increasingly prevalent during the final years of medical training to produce more competent doctors. Early exposure to SimMan can not only aid in acquisition of basic clinical skills but also prime students for later encounters with the modality to refine and acquire advanced clinical skills.

Limitations of the study include a single cohort of students with relatively small number in a single medical school. This study involved the students who were fast track entrants of the four year Accelerated MBBS programme and not the standard medical students. However, the session was conducted at the end of their preclinical year and just before entering clinical years, by which stage the level of knowledge and skills acquired are similar to the standard medical students of the five year programme. SimMan, a simulated patient in a clinical skills lab is not intended to and never will replace the learning that is derived from real patients in the clinical setting. However, simulation will enable preclinical medical students to establish a foundation in a range of clinical skills through experiential learning which eventually is developed in clinical practice.

Future work will seek to involve bigger cohort of students and also students from other institutions. Simulators are being used to assess medical students. Using SimMan for OSCE exams to test preclinical medical students can be explored in the future.

## Conclusions

To the best of our knowledge, this study represents the first to investigate the role of SimMan in clinical skills teaching for preclinical medical students. This study suggests that SimMan improves medical students’ ability to perform respiratory and cardiovascular examination on each other and; increases their knowledge and self- confidence to perform these skills in their preclinical years. All students strongly agreed that the session was useful. Thus SimMan can be a useful adjunct to facilitate student’s ability to acquire clinical skills effectively in preclinical years. Integrated learning by using SimMan as an adjunct to peer examination can be exciting and motivating in ways that might benefit learning by bridging the gap between preclinical and clinical years. It can increase the student’s awareness of the importance of acquiring clinical skills in preclinical years of their training and render them to be more prepared when they encounter patients in clinical practice.

## Competing interests

The authors declare that they have no competing interests.

## Authors’ contributions

MS was involved in conception, design, acquisition, analysis, interpretation of data, drafting of manuscript and literature review. TCB, RHT and HS were involved in the design, acquisition and analysis of the data. RF was involved in conception, design, revising it critically and final approval of the manuscript. All authors read and approved the final manuscript.

## Pre-publication history

The pre-publication history for this paper can be accessed here:

http://www.biomedcentral.com/1472-6920/13/20/prepub

## Supplementary Material

Additional file 1 **Appendix 1.** A flow chart demonstrating the study design. Click here for file
