# Supplementary material for: Role of SimMan in teaching clinical skills to preclinical medical students
Source: BMC Med Educ. 2013 Feb 10;13:20. doi: 10.1186/1472-6920-13-20 (PMC3572432; doi:10.1186/1472-6920-13-20)
Supplement: Additional file 1 — Appendix 1. A flow chart demonstrating the study design. [file 1472-6920-13-20-S1.docx]

EXAMINATION OF RESPIRATORY SYSTEM

| **Technique of handwashing using alcohol gel** | 1  -5 |
| --- | --- |
| **Inspection:**  Respiratory rate  Chest symmetry  Finger clubbing, nicotine stains  Cyanosis, anaemia | 1  1  1  1  0 |
| JVP  Oedema | 1  1  0 |
| **Palpation:**  Cervical lymph nodes  Checks for position of trachea  Chest expansion  Vocal fremitus | 1  1  1  1  0 |
| **Percussion:**  Good technique  Percusses symmetrically and bilaterally | 1  1  0 |
| **Auscultation:**  Uses stethoscope appropriately  Listens symmetrically  Listens to axilla  Listens to bases | 1  1  1  1  0 |
| **Summary:**  Provides an accurate summary one minute from the end | 2  0 |
| Global assessment of performance (introduces self, etc) | 1  0 |
| **TOTAL SCORE** |  |
